# Alternative use of suvorexant (Belsomra^®^) for the prevention of alcohol drinking and seeking in rats with a history of alcohol dependence

**DOI:** 10.3389/fnbeh.2022.1085882

**Published:** 2022-12-22

**Authors:** Francisco J. Flores-Ramirez, Jessica M. Illenberger, Glenn E. Pascasio, Alessandra Matzeu, Barbara J. Mason, Rémi Martin-Fardon

**Affiliations:** Department of Molecular Medicine, The Scripps Research Institute, La Jolla, CA, United States

**Keywords:** alcohol use disorder, stress, reinstatement, dual orexin receptor antagonist, suvorexant

## Abstract

Alcohol use disorder (AUD) is one of the most treatment-resistant medical conditions globally. The orexin (Orx) system regulates diverse physiological processes, including stress, and is a system of interest for the development of pharmaceuticals to treat substance use disorders, particularly AUD. The present study tested the ability of the dual orexin receptor antagonist suvorexant (SUV), marketed by Merck as Belsomra^®^, for the treatment of insomnia, to decrease alcohol self-administration and the stress-induced reinstatement of alcohol-seeking behavior in male Wistar rats with a history of alcohol dependence. Rats were trained to orally self-administer 10% alcohol (30 min/day for 3 weeks) and were either made dependent via chronic intermittent alcohol vapor exposure (14 h ON, 10 h OFF) for 6 weeks or exposed to air (non-dependent). Starting on week 7, the effect of SUV (0–20 mg/kg, p.o.) was tested on alcohol self-administration at acute abstinence (8 h after vapor was turned OFF) twice weekly. A separate cohort of rats that were prepared in parallel was removed from alcohol vapor exposure and then subjected to extinction training for 14 sessions. Once extinction was achieved, the rats received SUV (0 and 5 mg/kg, p.o.) and were tested for the footshock stress-induced reinstatement of alcohol-seeking behavior. Suvorexant at 5, 10, and 20 mg/kg selectively decreased alcohol intake in dependent rats. Furthermore, 5 mg/kg SUV prevented the stress-induced reinstatement of alcohol-seeking behavior in dependent rats only. These results underscore the significance of targeting the Orx system for the treatment of substance use disorders generally and suggest that repurposing SUV could be an alternative approach for the treatment of AUD.

## Introduction

Alcohol use disorder (AUD) is the most prevalent substance use disorder, with 3 million deaths per year that are attributable to alcohol (Grant et al., 2004, 2015; Hunt et al., 2020). In the United States alone, it is the third leading cause of preventable death, and according to the 2019 National Survey on Drug Use and Health^1^, in the U.S., 14.5 million people aged 12 or older (5.3% of this age group) had AUD. This includes 9 million men (6.8% of men in this age group) and 5.5 million women (3.9% of women in this age group). Treatments for AUD include behavioral, psychosocial, and pharmacological approaches, with the goal of reducing drinking and achieving and maintaining long-term abstinence from alcohol use (Witkiewitz et al., 2019). Only three pharmacological compounds have been approved by the United States Food and Drug Administration (FDA) for the treatment of AUD: disulfiram (acetaldehyde dehydrogenase inhibitor), acamprosate (which may act as an *N*-methyl-D-aspartate receptor agonist), and naltrexone (non-specific opioid receptor antagonist; Rosner et al., 2010; Maisel et al., 2013; Witkiewitz et al., 2019). These medications are effective in a subset of AUD patients, and the development and application of these compounds have helped improve our understanding of AUD pharmacotherapeutics generally, but alternative pharmacological avenues must continue to be explored and tested to treat a greater proportion of AUD patients.

The orexin (Orx; also known as hypocretin) hypothalamic neuropeptide system has arisen as a system of interest to develop AUD-specific therapeutics (Moorman, 2018). Orexin has long been known to be involved in regulating a wide range of physiological processes, including arousal, feeding, energy expenditure, and stress-related behavior (de Lecea et al., 1998; Sakurai et al., 1998; Sutcliffe and de Lecea, 2000; Mieda and Yanagisawa, 2002; Berridge et al., 2010; Teske et al., 2010). More recently, the Orx system has been implicated in highly motivated behavior, in addition to negative aspects of behavior, which together may underlie problematic alcohol use (Lawrence, 2010; Mahler et al., 2012; Brown and Lawrence, 2013; Barson and Leibowitz, 2016; Walker and Lawrence, 2017; Moorman, 2018; Matzeu and Martin-Fardon, 2021). Although there is a dearth of clinical research, some evidence suggests dysregulation of the Orx system in patients with AUD. For example, individuals who were diagnosed with AUD exhibited high blood levels of Orx during early withdrawal (Bayerlein et al., 2011; Ziolkowski et al., 2016). Importantly, these elevated Orx levels correlated with stress-related symptomatology during that same time period of withdrawal (von der Goltz et al., 2011).

Most of our understanding of the Orx system and its specific contribution to alcohol dependence has come from preclinical studies. Orexin was shown to promote excessive alcohol drinking when injected in the nucleus accumbens core but not shell (Schneider et al., 2007; Brown et al., 2013), ventral tegmental area but not substantia nigra (Srinivasan et al., 2012), and the anterior but not posterior paraventricular thalamus (Barson et al., 2015). Orexin cells are strongly recruited during alcohol-seeking behavior. For example, in rodent models of AUD, an increase in the activation of Orx neurons was observed during the reinstatement of alcohol seeking that was induced by alcohol-associated discriminative cues (Dayas et al., 2008), discrete cues (Moorman et al., 2016), alcohol-associated contexts (Hamlin et al., 2007; Millan et al., 2010), and stress (yohimbine; Kastman et al., 2016).

The involvement of the Orx system in alcohol drinking and seeking has been confirmed by pharmacological manipulations of Orx receptor 1 (OrxR1) and OrxR2. The systemic blockade of OrxR1 decreased alcohol drinking in a two-bottle choice paradigm in high-alcohol-preferring Sprague Dawley rats (Moorman and Aston-Jones, 2009), decreased alcohol self-administration under an operant fixed-ratio schedule (Lawrence et al., 2006; Richards et al., 2008; Moorman et al., 2017), and decreased alcohol self-administration under a progressive-ratio schedule (Jupp et al., 2011). The systemic administration of an OrxR1 antagonist decreased alcohol-seeking behavior that was induced by alcohol-related stimuli (Lawrence et al., 2006; Martin-Fardon and Weiss, 2014; Moorman et al., 2017) and decreased the stress (yohimbine)-induced reinstatement of alcohol seeking (Richards et al., 2008). The peripheral administration of an OrxR2 antagonist reduced alcohol but not saccharin self-administration in rats (Shoblock et al., 2011). The systemic administration of a dual orexin receptor antagonist (DORA) decreased breakpoints and reduced alcohol consumption under a progressive ratio schedule in alcohol-preferring rats (Anderson et al., 2014).

Two general observations can be made based on the literature with regard to the relationship between the Orx system and alcohol dependence: (i) exposure to alcohol strongly recruits the Orx system, and (ii) blocking Orx receptors (OrxR1, OrxR2, and both) decreases alcohol use. Recent interest has been seen in repurposing the FDA-approved DORA suvorexant (SUV), marketed by Merck as Belsomra^®^, for the treatment of insomnia (James et al., 2017; Campbell et al., 2020b). In addition to reducing drug craving (primarily *via* OrxR1), SUV may have the additional benefit of indirectly reducing relapse risk by normalizing sleep disturbances (primarily via OrxR2) that are commonly observed in AUD patients (Koob and Colrain, 2020).

One of the features of AUD is the significant increase of alcohol consumption (heavy drinking) to relieve or avoid withdrawal symptoms (Koob and Colrain, 2020). Moreover, knowing that stress is a major factor that contributes to the chronic relapsing and compulsive nature of substance use disorder, including AUD (Stephens and Wand, 2012), the present study’s objectives were to test the effect of SUV on two aspects of AUD: (i) the increase in alcohol consumption in alcohol-dependent rats and (ii) the stress-induced reinstatement of alcohol seeking.

## Materials and methods

### Animals

A total of 32 male Wistar rats (Charles River Laboratories, Hollister, CA, USA), weighing 150–170 g upon arrival, were housed two per cage in a humidity- and temperature-controlled vivarium on a reverse 12/12 h light/dark cycle (lights OFF at 8:00 a.m., lights ON at 8:00 p.m.) with free access to food and water. Before beginning the experimental procedures, the rats were given 1 week to acclimate to the housing and handling conditions. All behavioral procedures were conducted during the dark cycle (i.e., between 8:00 a.m. and 8:00 p.m.). All animal procedures were conducted in strict adherence to the National Institutes of Health *Guide for the Care and Use of Laboratory Animals* (National Research Council, 2013) and *Animal Research: Reporting In Vivo Experiments Guidelines* (Percie du Sert et al., 2020). The animal procedures were approved by the Institutional Animal Care and Use Committee of The Scripps Research Institute.

### Drugs

Suvorexant (Belsomra^®^; Merck, Whitehouse Station, NJ, USA) pills (20 mg) were crushed and dissolved in a Vitamin E TPGS (D-α-Tocopherol polyethylene glycol 1,000 succinate; Mazuri, Richmond, IN, USA) 20% solution (Cox et al., 2010; Guo et al., 2013; Ehlers et al., 2020). Once homogenized, to maximize bioavailability of the compound, SUV was administered orally (p.o.) at doses of 0, 5, 10, and 20 mg/kg in a volume of 5 ml/kg. The 0 mg SUV dose consisted of only vehicle that was used to dissolve SUV.

### Alcohol self-administration training

**FIGURE 1:**
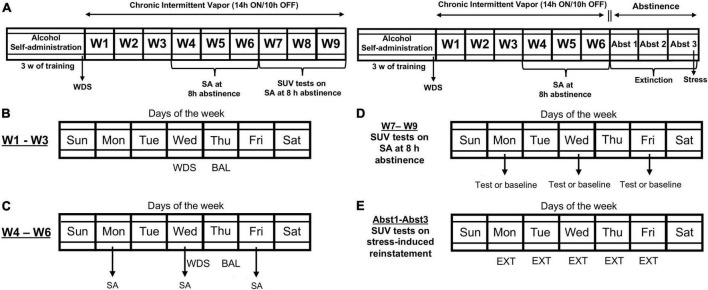
Timeline of the experimental procedures. **(A)** Male Wistar rats underwent 3 weeks of alcohol self-administration (SA) training. Upon the completion of training, baseline somatic withdrawal signs were recorded. **(B)** The rats were scored for somatic withdrawal signs during acute abstinence (8 h after the vapor was turned OFF on Wednesday), and blood alcohol levels were recorded 30 min before the alcohol vapors were turned OFF (on Thursdays) between weeks 1 and 3 of chronic intermittent alcohol vapor exposure. **(C)** The rats underwent self-administration sessions three times per week (Monday, Wednesday, and Friday) during acute abstinence (8 h after alcohol vapor was turned OFF) between weeks 4 and 6 of chronic intermittent alcohol vapor exposure. **(D)** Effect of SUV on alcohol self-administration. Between weeks 7 and 9, one group of rats was tested with all doses of SUV (0, 5, 10, and 20 mg/kg) at an acute abstinence point, in random order using a Latin-square design every other session. On days between testing, the rats underwent regular self-administration sessions, without SUV. **(E)** Effect of SUV on stress-induced reinstatement of alcohol-seeking behavior. After week 6 of dependence induction, a separate group of rats was removed from alcohol vapor exposure and underwent daily extinction sessions. Once extinction was achieved, the rats were tested for the intermittent footshock stress-induced reinstatement of alcohol-seeking behavior. BAL, blood alcohol level; EXT, extinction; SA, self-administration; SUV, suvorexant; WDS, somatic withdrawal signs; W, week; Abst, abstinence.

Alcohol self-administration training was conducted as previously reported (Matzeu and Martin-Fardon, 2020; Flores-Ramirez et al., 2022). Notably, no saccharin or sucrose fading procedure was required to induce voluntary alcohol intake. After the 1 week housing acclimation period and for the remainder of the training procedure (Figure 1A), the rats were given access to alcohol in standard operant conditioning chambers (29 × 24 × 19.5 cm; Med Associates, St. Albans, VT, USA) during daily 30 min self-administration sessions (for 3 weeks; Figure 1A) on a fixed-ratio 1 (FR1) schedule of reinforcement, in which responses on the right lever resulted in the delivery of 0.1 ml of 10% (w/v) alcohol (prepared in tap water from 95% w/v alcohol) and the brief (0.5 s) illumination of a cue light above the lever. Responses on the left inactive lever were recorded, but they resulted in no programed consequences.

### Chronic intermittent alcohol vapor exposure

Once self-administration training (21 sessions) was completed, half of the rats (*n* = 16) were made alcohol-dependent *via* chronic intermittent alcohol vapor exposure, and the other half were exposed to air only (*n* = 16; non-dependent group). During dependence induction (6 weeks; Figure 1A), the rats underwent daily cycles of 14 h of alcohol vapor ON and 10 h OFF. Blood alcohol levels (BALs) were measured using a gas chromatography-headspace blood analyzer (Agilent Technologies, Santa Clara, CA, USA). Blood alcohol levels (BALs) ranged between 150 and 250 mg%. For 3 weeks, the rats remained undisturbed, apart from measuring BALs during the last 30 min of vapor exposure (on Thursday) and scoring somatic signs of withdrawal (at 8 h of abstinence) once weekly (on Wednesday; Figure 1B). Behavioral signs of withdrawal were measured by a laboratory assistant who was blind to the experimental conditions using a scale that was adapted from an original report by Macey et al. (1996). These withdrawal signs included measures of ventromedial limb retraction, vocalization (i.e., irritability to touch), tail stiffness, abnormal gait, and body tremors. Each of these behaviors were assigned a score of 0–2, based on severity: 0 = no signs, 1 = moderate, and 2 = severe. To confirm alcohol dependence and assess withdrawal severity, the sum of the five scores (0–10) was used as a quantitative measure. This approach was used because this model of alcohol dependence is well-known to lead to motivational and somatic signs of withdrawal in rats (Vendruscolo and Roberts, 2014). Baseline withdrawal scores were measured before the last training session (Day 21). At weeks 4, 5, and 6 of alcohol vapor exposure (Figure 1C), the animals underwent 30 min FR1 alcohol self-administration sessions 8 h after the alcohol vapor was turned OFF and when blood and brain alcohol levels are negligible, three times per week (Monday, Wednesday, and Friday). Baseline self-administration levels were obtained by averaging the last three self-administration training sessions. Air-exposed animals (non-dependent) were subjected to the same BALs assessment, withdrawal testing, and alcohol self-administration sessions during weeks 4–6 as the dependent subjects.

### Effects of suv on alcohol self-administration

Starting on week 7 of chronic intermittent alcohol vapor exposure (Figure 1A, Left panel), the effects of SUV (0, 5, 10, and 20 mg/kg) on alcohol self-administration were evaluated in half of the rats (*n* = 16; Figure 1D). Suvorexant was administered orally (p.o.) 30 min before the start of the self-administration sessions at an acute abstinence point (8 h after the alcohol vapor was turned OFF). To control for possible order effects of SUV dosing on self-administration, each animal was tested with all doses of SUV in random order using a Latin-square design every other session. On days between testing, the rats underwent regular self-administration sessions, without pharmacological administration.

### Extinction training and stress-induced reinstatement

The other half of the rats (*n* = 16) that were used for the stress-induced reinstatement experiment and were prepared in parallel (see Figure 1A, Right panel). After the 6 weeks of chronic intermittent alcohol vapor exposure, the rats were removed from the alcohol vapor chambers and started a 3 weeks abstinence period (Figure 1A, Right panel). During these 3 weeks, the rats underwent extinction training (30 min session, five times/week, for a total of 14 sessions over 3 weeks; Figure 1E). These extinction sessions were identical to the alcohol self-administration sessions, but alcohol was withheld. For habituation to the footshock stress procedure, the rats were placed in the operant chambers 15 min before each session. At the end of this 15 min period, both levers were extended into the operant chambers, and the extinction session began.

Twenty-four hours after the last extinction training session, the rats were tested for the reinstatement of footshock stress-induced alcohol-seeking behavior (Figure 1A, Right panel). Specifically, 30 min before testing, the rats were given SUV (0 or 5 mg/kg, p.o.) and left undisturbed until placed in the operant chambers and subjected to footshock stress (15 min; variable intermittent electric footshock, 0.5 mA; duration, 0.5 s; mean shock interval, 40 s; (Martin-Fardon et al., 2000; Zhao et al., 2006; Sidhpura et al., 2010; Matzeu and Martin-Fardon, 2020; Flores-Ramirez et al., 2022). Two minutes after the termination of footshock, the levers were extended into the chamber, and responses were recorded for 30 min. Each animal was tested only once with vehicle or 5 mg/kg SUV according to a between-subjects design. The 5 mg SUV dose was selected because it was found to be the lowest effective dose at reducing alcohol self-administration in the alcohol-dependent group of rats.

### Statistical analysis

The acquisition of alcohol self-administration during the 3 weeks of training was analyzed using a two-way repeated-measures analysis of variance (ANOVA), with session and lever (i.e., active vs. inactive) as within- and between-subjects factors, respectively. Total alcohol intake (g/kg) during self-administration training was analyzed using a one-way repeated-measures ANOVA. Self-administration during chronic intermittent alcohol vapor exposure (i.e., baseline vs. weeks 4, 5, and 6) was analyzed using two-way repeated-measures ANOVA, with time and alcohol dependence as independent factors. Chronic intermittent alcohol vapor exposure’s effect on somatic withdrawal signs was analyzed using a Kruskal-Wallis test, followed by Dunn’s tests. The effect of SUV on alcohol self-administration was analyzed using two-way repeated-measures ANOVA, with alcohol dependence and treatment (i.e., 0 vs. 5, 10, and 20 mg/kg SUV) as sources of variance. Finally, stress-induced reinstatement was analyzed using a two-way ANOVA, with alcohol dependence (i.e., non-dependent vs. dependent) and treatment (i.e., 0 vs. 5 mg/kg SUV) as independent factors. Significant interactions and main effects in the ANOVAs were followed by the Tukey *post-hoc* test. The data are expressed as the mean + SEM. Values of *p* < 0.05 were considered statistically significant. The statistical analyses were performed using Prism 8 software (GraphPad, San Diego, CA, USA).

## Results

### Alcohol self-administration training and escalation

**FIGURE 2:**
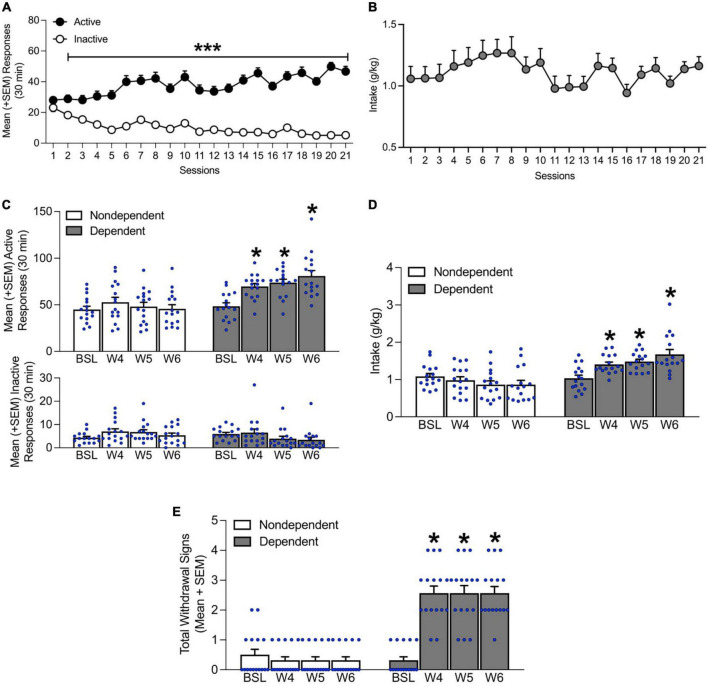
Time course of alcohol self-administration acquisition during the 3 weeks of training and escalation of drinking during weeks 4–6 of chronic intermittent alcohol vapor exposure. **(A)** Rats acquired self-administration over the 21 training sessions. **(B)** Alcohol intake remained stable throughout the 21 training sessions. **(C)** At weeks 4–6 of chronic intermittent alcohol vapor exposure, the alcohol-dependent group of rats exhibited a significant increase in the number of responses on the active lever. No differences in inactive lever responses were observed. **(D)** Alcohol intake also increased during weeks 4–6 of intermittent alcohol vapor exposure. **(E)** An increase in somatic withdrawal signs was observed in dependent rats on weeks 4–6 of chronic intermittent alcohol vapor exposure during acute abstinence. The data are expressed as mean + SEM. ***p < 0.05, vs. inactive lever; *p < 0.05, vs. respective baseline. BSL, baseline; W, week.

Over 21 sessions of training (30 min/day), all the rats acquired alcohol self-administration (two-way repeated-measures ANOVA; time: *F*_1,651_ = 1546, *p* < 0.05; lever: *F*_20,651_ = 2.01, *p* < 0.05; time × lever interaction: *F*_20,651_ = 9.67, *p* < 0.05; Figure 2A). Tukey’s multiple-comparison *post hoc* test confirmed that active lever presses were significantly higher than inactive lever presses starting in session 2 (*p* < 0.05). Additionally, overall intake remained stable throughout the 21 training sessions (*p* > 0.05; Figure 2B).

During weeks 4, 5, and 6 of chronic intermittent alcohol vapor exposure, alcohol-dependent rats exhibited an increase in the number of responses on the active lever (*p* < 0.05, Tukey *post-hoc* tests vs. baseline following two-way repeated-measures ANOVA; time: *F*_3,90_ = 9.81, *p* < 0.05; alcohol dependence: *F*_1,30_ = 19.69, *p* < 0.05; time × alcohol dependence interaction: *F*_3,90_ = 7.69, *p* < 0.05; Figure 2C). No differences in inactive lever responses were observed (*p* > 0.05; Figure 2C). Alcohol intake, a measure that was obtained by averaging the intake data that were recorded Monday, Wednesday, and Friday of that week also increased (*p* < 0.05, Tukey *post-hoc* tests vs. baseline following two-way repeated-measures ANOVA; time: *F*_3,90_ = 2.95, *p* < 0.05; alcohol dependence: *F*_1,30_ = 21.15, *p* < 0.05; time × alcohol dependence interaction: *F*_3,90_ = 13.19, *p* < 0.05; Figure 2D).

During weeks 4, 5, and 6, alcohol-dependent rats exhibited significantly higher somatic withdrawal signs at an acute abstinence point (8 h after vapors were off; *p* < 0.05, Dunn’s test vs. baseline following Kruskal-Wallis non-parametric test: χ^2^(7) = 88.69, *p* < 0.05; Figure 2E).

### Effects of suv on alcohol intake

**FIGURE 3:**
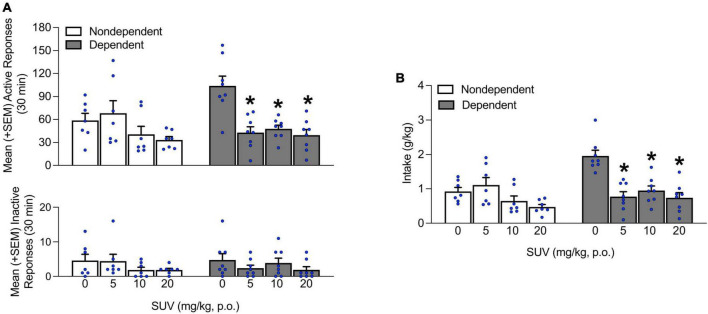
Effect of SUV (0, 5, 10, and 20 mg/kg) on alcohol self-administration. **(A)** The administration of SUV (5, 10, and 20 mg/kg) significantly decreased the number of responses at the active lever in dependent rats at all doses tested and did not produce any effects in non-dependent rats. No differences in inactive lever responses were observed. **(B)** Similarly, intake was reduced in dependent rats at all doses tested and did not produce any effects in non-dependent rats. The data are expressed as the mean + SEM. *p < 0.05, vs. 0 mg/kg. SUV, suvorexant.

After the 6 weeks of dependence induction, the ability of SUV to reduce alcohol self-administration was evaluated. In non-dependent rats, SUV pretreatment did not affect alcohol self-administration, regardless of dose. However, SUV administration significantly decreased the number of responses at the active lever at the 5, 10, and 20 mg/kg doses in alcohol-dependent rats (*p* < 0.05, Tukey *post-hoc* tests vs. vehicle following two-way repeated-measures ANOVA; dose: *F*_3,39_ = 44.33, *p* < 0.05; alcohol dependence × dose: *F*_3,39_ = 5.85, *p* < 0.05; Figure 3A). Importantly, no differences in inactive lever responses were observed, regardless of that rats’ history of alcohol dependence and/or SUV dose (*p* > 0.05; Figure 3A). The effect of SUV was similar when assessing alcohol intake (*p* < 0.05, Tukey *post-hoc* tests vs. vehicle following two-way repeated-measures ANOVA; alcohol dependence: *F*_1,13_ = 5.03, *p* < 0.05; dose: *F*_3,39_ = 15.46, *p* < 0.05; alcohol dependence × dose: *F*_3,39_ = 9.62, *p* < 0.05; Figure 3B).

### Stress-induced reinstatement

**FIGURE 4:**
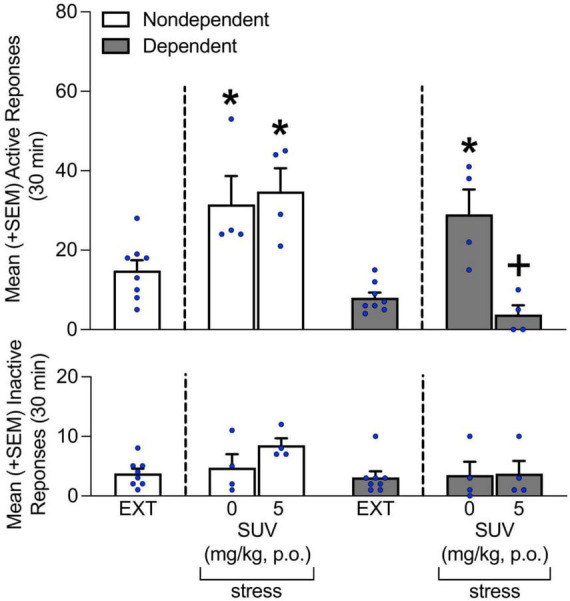
Effect of SUV (0 and 5 mg/kg) on stress-induced reinstatement of alcohol-seeking behavior. Intermittent footshock stress induced alcohol-seeking behavior in rats that received vehicle (0 mg/kg) in both the dependent and non-dependent groups. The administration of SUV prevented the stress-induced reinstatement of alcohol seeking in dependent rats but not in non-dependent rats. No differences in inactive lever responses were observed. The data are expressed as the mean + SEM. *p < 0.05, vs. respective EXT; ^+^*p* < 0.05, vs. respective 5 mg/kg SUV. EXT, extinction; SUV, suvorexant.

Under vehicle condition (i.e., 0 mg/kg SUV; Figure 4) stress precipitated the reinstatement of alcohol-seeking behavior in both non-dependent and dependent rats. Of note, even though the reinstatement of alcohol-seeking observed in both groups was similar, it was was prevented by the administration of 5 mg/kg SUV only in rats with a history of alcohol dependence (Tukey *post-hoc* test following two-way ANOVA; treatment: *F*_1,13_ = 11.69, *p* < 0.05; alcohol dependence: *F*_2,13_ = 20.62, *p* < 0.05; treatment × alcohol dependence interaction: *F*_2,13_ = 4.52, *p* < 0.05; Figure 4). No differences in inactive lever responses were observed, regardless of the rats’ history of alcohol dependence and/or treatment condition (*p* > 0.05; Figure 4).

## Discussion

The present study assessed the ability of SUV, an FDA-approved DORA for the treatment of insomnia, to be repurposed to reduce alcohol self-administration in animals with a history of alcohol dependence. This study also tested SUV’s ability to decrease the stress-induced reinstatement of alcohol-seeking behavior in alcohol-dependent rats. Alcohol-dependent rats exhibited an increase in alcohol self-administration (i.e., escalation) during alcohol dependence that was induced by chronic intermittent alcohol vapor exposure, which is consistent with several previous studies (O’Dell et al., 2004; Vendruscolo and Roberts, 2014; Matzeu et al., 2018a; Matzeu and Martin-Fardon, 2020). In agreement with previous reports, exposure to intermittent footshock stress reliably reinstated previously extinguished alcohol-seeking behavior (Le et al., 1999; Martin-Fardon et al., 2000; Matzeu and Martin-Fardon, 2020; Flores-Ramirez et al., 2022). Notably, SUV significantly reduced alcohol self-administration only in alcohol-dependent rats. Furthermore, SUV selectively blocked the stress-induced reinstatement of alcohol-seeking behavior in alcohol-dependent rats. These results support the hypothesis that both OrxR1 and OrxR2 play significant roles in drug self-administration and the reinstatement of drug-seeking behavior (Plaza-Zabala et al., 2012, 2013; Uslaner et al., 2014), further implicating the Orx system in maladaptive motivation, reflected by an increase in the motivation to drink and seek alcohol during dependence (Moorman and Aston-Jones, 2009; Moorman et al., 2017). Altogether, the results highlight the significance of targeting the Orx system for the treatment of substance use disorders, and SUV could be repurposed for the treatment of AUD.

Alcohol-dependent rats in the present study exhibited an increase in alcohol self-administration and greater somatic withdrawal signs during weeks 4–6 of intermittent alcohol vapor exposure. These results mirror findings that rats with a history of alcohol dependence exhibit an increase in alcohol self-administration (i.e., escalation) and somatic and motivational signs of withdrawal that are characteristic of dependence, which are observable at 6–8 h of abstinence from alcohol vapor (Roberts et al., 1996; O’Dell et al., 2004; Vendruscolo and Roberts, 2014; Matzeu et al., 2018a; Matzeu and Martin-Fardon, 2020). The present results add to the growing body of literature that shows that intermittent exposure to alcohol vapor elicits behavioral and neurobiological signs of dependence. One explanation is that these changes in neurobehavioral systems, especially those that underlie response inhibition and reward- and stress-related behavior, contribute to hyperkatifeia and thus negative reinforcement, whereby dependent subjects seek relief from negative symptoms that are exacerbated during alcohol withdrawal, further motivating them to seek and take alcohol, particularly during times of heightened stress (Koob, 2014).

The present results showed that 5, 10, and 20 mg/kg SUV significantly and selectively decreased alcohol self-administration in rats with a history of alcohol dependence, without affecting alcohol intake in non-dependent rats. These results are consistent with previous reports that manipulations of the Orx system, *via* the pharmacological blockade of OrxR1 and OrxR2, influence alcohol consumption in rodents (for review, see Kim et al., 2012). Indeed, systemic administration of the OrxR1 antagonist SB334867 decreased voluntary home-cage alcohol consumption (Moorman and Aston-Jones, 2009; Anderson et al., 2014) and reduced operant self-administration (Lawrence et al., 2006; Richards et al., 2008; Jupp et al., 2011). Similarly, the OrxR2 antagonist LSN2424100 decreased home-cage alcohol consumption in alcohol-preferring (P) rats and alcohol-preferring C57BL/6J mice (Anderson et al., 2014). Furthermore, the DORA almorexant (ACT-078573) reduced alcohol self-administration under both fixed- and progressive-ratio schedules of reinforcement and reduced home-cage alcohol drinking in rats and mice (Srinivasan et al., 2012; Anderson et al., 2014). The present findings, together with the extant literature, provide further evidence that the Orx system is a prominent player in the increase in alcohol consumption under conditions of alcohol dependence. The data further suggest that the Orx system is dysregulated as a function of alcohol exposure, demonstrated by the selective effect of SUV to decrease alcohol self-administration only in alcohol-dependent rats.

Intermittent footshock stress induced alcohol-seeking behavior in non-dependent and dependent rats under the vehicle condition (i.e., 0 mg/kg SUV), at a similar magnitude, even though alcohol dependent rats displayed greater active lever responses during dependence induction ($\bar{X}$ = 81 ± 5 vs. $\bar{X}$ = 46 ± 4, respectively at week 6). Importantly, 5 mg/kg SUV prevented the stress-induced reinstatement of alcohol-seeking behavior in dependent rats only. In agreement with the present findings, previous studies showed that peripheral (Martin-Fardon and Weiss, 2014) and central (intra-prelimbic cortex) injections of the OrxR1 antagonist SB334867 decreased reinstatement that was induced by alcohol-related stimuli (Martin-Fardon and Weiss, 2014; Brown et al., 2016), and that systemic SB334867 administration also reduced yohimbine (stress) induced reinstatement (Richards et al., 2008). More recently, an injection of the DORA TCS1102 in the posterior paraventricular nucleus of the thalamus prevented the footshock stress-induced reinstatement of alcohol-seeking behavior in alcohol-dependent rats only (Matzeu and Martin-Fardon, 2020). Altogether, these findings indicate that the Orx system plays a pivotal role in motivational aspects of alcohol-seeking behavior, and the antagonism of its target receptors may be an effective treatment to reverse Orx dysregulation that is induced by exposure to alcohol. Dysregulation of the Orx system by chronic alcohol is consistent with clinical observations in individuals with AUD who present high plasma Orx levels during acute withdrawal (Bayerlein et al., 2011; Ziolkowski et al., 2016) that were correlated with exacerbations in affect- and stress-related symptomatology (von der Goltz et al., 2011).

An interesting finding was that SUV administration selectively decreased alcohol taking and seeking in alcohol-dependent rats. A recurring theme in the expanding Orx literature is that the pharmacological blockade of OrxRs is more effective in subjects with high motivation for alcohol seeking or when alcohol drinking is exacerbated (i.e., alcohol preference or dependence induction). For example, the administration of an OrxR1 antagonist decreased alcohol self-administration and reinstatement behavior in rats that were trained to exhibit high motivation for alcohol (Moorman and Aston-Jones, 2009; Moorman et al., 2017) and in rats that were bred for high alcohol preference (Lawrence et al., 2006; Dhaher et al., 2010; Anderson et al., 2014). OrxR1 blockade selectively decreased escalated alcohol drinking in dependent but not non-dependent mice (Lopez et al., 2016), and blockade of both OrxR1 and OrxR2 decreased alcohol drinking in dependent rats in agreement with the present study (Aldridge et al., 2022). Ultimately, two recent studies from our group (Matzeu and Martin-Fardon, 2020; Flores-Ramirez et al., 2022) also found that the effects of a DORA on alcohol-seeking behavior are more robust in dependent animals than in non-dependent animals. A possible explanation for this phenomenon is that the unique contribution of Orx transmission to motivational aspects of alcohol taking and stress-induced alcohol seeking does not play a significant role until anti-reward systems are sufficiently engaged or recruited. Thus, in animals that are highly motivated to consume alcohol (e.g., alcohol-dependent animals), Orx transmission is potentially compromised and promotes the incentive for alcohol drinking and seeking through negative reinforcement mechanisms. Collectively, these findings may have significant clinical implications. Treatment with SUV may be beneficial for decreasing alcohol craving and relapse in individuals who have been diagnosed with AUD.

Although Orx is exclusively produced in the hypothalamus (HYP), including the lateral HYP, dorsomedial HYP, and perifornical area (Baldo et al., 2003; DiLeone et al., 2003; Winsky-Sommerer et al., 2004), Orx neurons project throughout the brain, densely innervating an array of brain regions that are involved in arousal, motivation, and responsivity to stress-related stimuli (Peyron et al., 1998; Baldo et al., 2003; Grafe and Bhatnagar, 2018). Although systemic approaches to understand how the Orx system influences motivational processes that underlie compulsive alcohol taking and seeking have been successful, it is important to also uncover unique contributions of discreet brain regions. Previous research showed that OrxR1 blockade in the ventral tegmental area (VTA) decreased the cue-induced reinstatement of alcohol seeking (Brown et al., 2016), and that intra-VTA DORA administration decreased alcohol self-administration (Srinivasan et al., 2012). Furthermore, targeted OrxR1 blockade in the medial prefrontal cortex decreased the cue-induced reinstatement of responding for alcohol, and the blockade of OrxR1 in the nucleus accumbens shell decreased alcohol self-administration (Lei et al., 2016), and decreased alcohol-seeking when the blockade occurred in the lateral HYP (Campbell et al., 2020a). The direct administration of a OrxR2 antagonist in the central nucleus of the amygdala decreased alcohol intake in mice (Olney et al., 2017), whereas an infusion of the DORA TCS1102 in the posterior paraventricular nucleus of the thalamus prevented the stress-induced reinstatement of alcohol-seeking behavior (Matzeu and Martin-Fardon, 2020). These findings suggest that Orx system activity throughout a wide system of brain regions is key in mediating behaviors that are related to alcohol taking and seeking. The present study administered SUV only systemically, but the brain regions mentioned above likely play a significant role, which requires further testing to delineate the exact anatomical and network bases of the behavioral effects of SUV.

One limitation of the present study was that female rats were not included in our experimental design, which limits generalizability of the results. The literature shows well-established sex differences in alcohol intake and preference in two-bottle choice tests (Li and Lumeng, 1984; Blanchard et al., 1993; Walker et al., 2008), differences in BALs after self-administration and somatic withdrawal signs during intermittent alcohol vapor exposure (Matzeu et al., 2018b), and differences in reactivity to rewarding and aversive properties of alcohol (Torres et al., 2014). Furthermore, orexins have also been found to mediate sex-dependent effects in stress responsivity (Grafe et al., 2017). Future studies should elucidate possible sex-specific effects of SUV on alcohol taking and seeking. Another possible limitation of this study is the relatively small number of rats used for testing SUV on the stress induced-reinstatement of alcohol-seeking behavior (*n* = 4/dose). Although the probability exists that a substantially larger cohort of rats might yield a different result, previous research from our laboratory argues against this possibility. In fact, it was shown that TCS1102, another DORA is more efficacious at reducing stress-induced reinstatement of alcohol-seeking behavior in rats with a history of alcohol dependence (Matzeu and Martin-Fardon, 2020; Flores-Ramirez et al., 2022), strongly supporting the beneficial effects of targeting both OrxR1 and OrxR2 to prevent stress-induced craving and relapse in individuals suffering form AUD. Individuals who are prescribed SUV for the treatment of insomnia are taking the medication before bedtime and are advised not to consume alcohol because of their possible additive effects, which may result in increased risk of somnolence (Sun et al., 2015). Therefore, an important concern will be the time during the day when SUV is administered to be the most efficacious at treating AUD and minimize daytime somnolence. In this context, several clinical trials with SUV have been initiated (i.e., ClinicalTrials.gov Identifier: NCT04229095 and NCT03897062) and further clinical research is warranted to determine the safest approach to administer SUV to patients suffering of AUD.

In summary, the present findings demonstrate that the administration of SUV, a currently FDA-approved treatment for insomnia, selectively decreased alcohol self-administration and the stress-induced reinstatement of alcohol-seeking behavior in animals with a history of dependence. The present results highlight the significance of targeting the Orx system for the treatment of substance use disorders and suggest that repurposing SUV could be a good alternative for the treatment of AUD to prevent compulsive-like drinking and stress-induced craving and relapse.

## Data availability statement

The original contributions presented in this study are included in the article/supplementary materials, further inquiries can be directed to the corresponding author.

## Ethics statement

The animal study was reviewed and approved by Institutional Animal Care and Use Committee of The Scripps Research Institute.

## Author contributions

RM-F conceived the study. RM-F and BM designed the research. FF-R, JI, GP, and AM performed the experimental procedures, analyzed the results, and created the figures. All authors co-wrote the manuscript, contributed to data interpretation, and manuscript editing and approved the submitted version.

## Footnotes

1
https://www.niaaa.nih.gov

## References

[B1] Aldridge G. M. Zarin T. A. Brandner A. J. George O. Gilpin N. W. Repunte-Canonigo V. (2022). Effects of single and dual hypocretin-receptor blockade or knockdown of hypocretin projections to the central amygdala on alcohol drinking in dependent male rats. *Addict. Neurosci.* 3:100028. 10.1016/j.addicn.2022.100028 35965958 PMC9365098

[B2] Anderson R. I. Becker H. C. Adams B. L. Jesudason C. D. Rorick-Kehn L. M. (2014). Orexin-1 and orexin-2 receptor antagonists reduce ethanol self-administration in high-drinking rodent models. *Front. Neurosci.* 8:33. 10.3389/fnins.2014.00033 24616657 PMC3933945

[B3] Baldo B. A. Daniel R. A. Berridge C. W. Kelley A. E. (2003). Overlapping distributions of orexin/hypocretin- and dopamine-beta-hydroxylase immunoreactive fibers in rat brain regions mediating arousal, motivation, and stress. *J. Comp. Neurol.* 464 220–237. 10.1002/cne.10783 12898614

[B4] Barson J. R. Leibowitz S. F. (2016). Hypothalamic neuropeptide signaling in alcohol addiction. *Prog. Neuropsychopharmacol. Biol. Psychiatry* 65 321–329. 10.1016/j.pnpbp.2015.02.006 25689818 PMC4537397

[B5] Barson J. R. Ho H. T. Leibowitz S. F. (2015). Anterior thalamic paraventricular nucleus is involved in intermittent access ethanol drinking: Role of orexin receptor 2. *Addict. Biol.* 20 469–481. 10.1111/adb.12139 24712379 PMC4192116

[B6] Bayerlein K. Kraus T. Leinonen I. Pilniok D. Rotter A. Hofner B. (2011). Orexin A expression and promoter methylation in patients with alcohol dependence comparing acute and protracted withdrawal. *Alcohol* 45 541–547. 10.1016/j.alcohol.2011.02.306 21621370

[B7] Berridge C. W. Espana R. A. Vittoz N. M. (2010). Hypocretin/orexin in arousal and stress. *Brain Res.* 1314 91–102. 10.1016/j.brainres.2009.09.019 19748490 PMC2819651

[B8] Blanchard B. A. Steindorf S. Wang S. LeFevre R. Mankes R. F. Glick S. D. (1993). Prenatal ethanol exposure alters ethanol-induced dopamine release in nucleus accumbens and striatum in male and female rats. *Alcohol Clin. Exp. Res.* 17 974–981. 10.1111/j.1530-0277.1993.tb05651.x 8279684

[B9] Brown R. M. Lawrence A. J. (2013). Ascending orexinergic pathways and alcohol-seeking. *Curr. Opin. Neurobiol.* 23 467–472. 10.1016/j.conb.2013.02.014 23537903

[B10] Brown R. M. Khoo S. Y. Lawrence A. J. (2013). Central orexin (hypocretin) 2 receptor antagonism reduces ethanol self-administration, but not cue-conditioned ethanol-seeking, in ethanol-preferring rats. *Int. J. Neuropsychopharmacol.* 16 2067–2079. 10.1017/S1461145713000333 23601187

[B11] Brown R. M. Kim A. K. Khoo S. Y. Kim J. H. Jupp B. Lawrence A. J. (2016). Orexin-1 receptor signalling in the prelimbic cortex and ventral tegmental area regulates cue-induced reinstatement of ethanol-seeking in iP rats. *Addict. Biol.* 21 603–612. 10.1111/adb.12251 25899624

[B12] Campbell E. J. Hill M. K. Maddern X. J. Jin S. Pang T. Y. Lawrence A. J. (2020a). Orexin-1 receptor signaling within the lateral hypothalamus, but not bed nucleus of the stria terminalis, mediates context-induced relapse to alcohol seeking. *J. Psychopharmacol.* 34 1261–1270. 10.1177/0269881120959638 33063594

[B13] Campbell E. J. Marchant N. J. Lawrence A. J. (2020b). A sleeping giant: Suvorexant for the treatment of alcohol use disorder? *Brain Res.* 1731:145902. 10.1016/j.brainres.2018.08.005 30081035

[B14] Cox C. D. Breslin M. J. Whitman D. B. Schreier J. D. McGaughey G. B. Bogusky M. J. (2010). Discovery of the dual orexin receptor antagonist [(7R)-4-(5-chloro-1,3-benzoxazol-2-yl)-7-methyl-1,4-diazepan-1-yl][5-methyl-2-(2H -1,2,3-triazol-2-yl)phenyl]methanone (MK-4305) for the treatment of insomnia. *J. Med. Chem.* 53 5320–5332. 10.1021/jm100541c 20565075

[B15] Dayas C. V. McGranahan T. M. Martin-Fardon R. Weiss F. (2008). Stimuli linked to ethanol availability activate hypothalamic CART and orexin neurons in a reinstatement model of relapse. *Biol. Psychiatry* 63 152–157. 10.1016/j.biopsych.2007.02.002 17570346

[B16] de Lecea L. Kilduff T. S. Peyron C. Gao X. Foye P. E. Danielson P. E. (1998). The hypocretins: Hypothalamus-specific peptides with neuroexcitatory activity. *Proc. Natl. Acad. Sci. U.S.A.* 95 322–327. 10.1073/pnas.95.1.322 9419374 PMC18213

[B17] Dhaher R. Hauser S. R. Getachew B. Bell R. L. McBride W. J. McKinzie D. L. (2010). The Orexin-1 receptor antagonist SB-334867 reduces alcohol relapse drinking, but not alcohol-seeking, in alcohol-preferring (P) rats. *J. Addict. Med.* 4 153–159. 10.1097/ADM.0b013e3181bd893f 20871792 PMC2943642

[B18] DiLeone R. J. Georgescu D. Nestler E. J. (2003). Lateral hypothalamic neuropeptides in reward and drug addiction. *Life Sci.* 73 759–768. 10.1016/s0024-3205(03)00408-9 12801597

[B19] Ehlers C. L. Benedict J. Wills D. Sanchez-Alavez M. (2020). PSPH-D-18-00526: Effect of a dual orexin receptor antagonist (DORA-12) on sleep and event-related oscillations in rats exposed to ethanol vapor during adolescence. *Psychopharmacology (Berl)* 237 2917–2927. 10.1007/s00213-019-05371-4 31659377 PMC7186151

[B20] Flores-Ramirez F. J. Matzeu A. Sanchez-Marin L. Martin-Fardon R. (2022). Blockade of corticotropin-releasing factor-1 receptors in the infralimbic cortex prevents stress-induced reinstatement of alcohol seeking in male Wistar rats: Evidence of interaction between CRF1 and orexin receptor signaling. *Neuropharmacology* 210:109046. 10.1016/j.neuropharm.2022.109046 35341789 PMC9176217

[B21] Grafe L. A. Bhatnagar S. (2018). Orexins and stress. *Front. Neuroendocrinol.* 51:132–145. 10.1016/j.yfrne.2018.06.003 29932958 PMC6345253

[B22] Grafe L. A. Cornfeld A. Luz S. Valentino R. Bhatnagar S. (2017). Orexins mediate sex differences in the stress response and in cognitive flexibility. *Biol. Psychiatry* 81 683–692. 10.1016/j.biopsych.2016.10.013 27955897 PMC5359079

[B23] Grant B. F. Goldstein R. B. Saha T. D. Chou S. P. Jung J. Zhang H. (2015). Epidemiology of DSM-5 alcohol use disorder: Results from the national epidemiologic survey on alcohol and related conditions III. *JAMA Psychiatry* 72 757–766. 10.1001/jamapsychiatry.2015.0584 26039070 PMC5240584

[B24] Grant B. F. Stinson F. S. Dawson D. A. Chou S. P. Dufour M. C. Compton W. (2004). Prevalence and co-occurrence of substance use disorders and independent mood and anxiety disorders: Results from the National Epidemiologic Survey on Alcohol and Related Conditions. *Arch. Gen. Psychiatry* 61 807–816. 10.1001/archpsyc.61.8.807 15289279

[B25] Guo Y. Luo J. Tan S. Otieno B. O. Zhang Z. (2013). The applications of Vitamin E TPGS in drug delivery. *Eur. J. Pharm. Sci.* 49 175–186. 10.1016/j.ejps.2013.02.006 23485439

[B26] Hamlin A. S. Newby J. McNally G. P. (2007). The neural correlates and role of D1 dopamine receptors in renewal of extinguished alcohol-seeking. *Neuroscience* 146 525–536. 10.1016/j.neuroscience.2007.01.063 17360123

[B27] Hunt G. E. Malhi G. S. Lai H. M. X. Cleary M. (2020). Prevalence of comorbid substance use in major depressive disorder in community and clinical settings, 1990-2019: Systematic review and meta-analysis. *J. Affect. Disord.* 266 288–304. 10.1016/j.jad.2020.01.141 32056890

[B28] James M. H. Mahler S. V. Moorman D. E. Aston-Jones G. (2017). A decade of orexin/hypocretin and addiction: Where are we now? *Curr. Top. Behav. Neurosci.* 33 247–281. 10.1007/7854_2016_57 28012090 PMC5799809

[B29] Jupp B. Krivdic B. Krstew E. Lawrence A. J. (2011). The orexin(1) receptor antagonist SB-334867 dissociates the motivational properties of alcohol and sucrose in rats. *Brain Res.* 1391 54–59. 10.1016/j.brainres.2011.03.045 21439948

[B30] Kastman H. E. Blasiak A. Walker L. Siwiec M. Krstew E. V. Gundlach A. L. (2016). Nucleus incertus Orexin2 receptors mediate alcohol seeking in rats. *Neuropharmacology* 110(Pt A) 82–91. 10.1016/j.neuropharm.2016.07.006 27395787

[B31] Kim A. K. Brown R. M. Lawrence A. J. (2012). The role of orexins/hypocretins in alcohol use and abuse: An appetitive-reward relationship. *Front. Behav. Neurosci.* 6:78. 10.3389/fnbeh.2012.00078 23189046 PMC3504295

[B32] Koob G. F. (2014). Neurocircuitry of alcohol addiction: Synthesis from animal models. *Handb. Clin. Neurol.* 125 33–54. 10.1016/B978-0-444-62619-6.00003-3 25307567

[B33] Koob G. F. Colrain I. M. (2020). Alcohol use disorder and sleep disturbances: A feed-forward allostatic framework. *Neuropsychopharmacology* 45 141–165. 10.1038/s41386-019-0446-0 31234199 PMC6879503

[B34] Lawrence A. J. (2010). Regulation of alcohol-seeking by orexin (hypocretin) neurons. *Brain Res.* 1314 124–129. 10.1016/j.brainres.2009.07.072 19646424

[B35] Lawrence A. J. Cowen M. S. Yang H. J. Chen F. Oldfield B. (2006). The orexin system regulates alcohol-seeking in rats. *Br. J. Pharmacol.* 148 752–759. 10.1038/sj.bjp.0706789 16751790 PMC1617074

[B36] Le A. D. Poulos C. X. Harding S. Watchus J. Juzytsch W. Shaham Y. (1999). Effects of naltrexone and fluoxetine on alcohol self-administration and reinstatement of alcohol seeking induced by priming injections of alcohol and exposure to stress. *Neuropsychopharmacology* 21 435–444. 10.1016/S0893-133X(99)00024-X 10457541

[B37] Lei K. Wegner S. A. Yu J. H. Mototake A. Hu B. Hopf F. W. (2016). Nucleus accumbens Shell and mPFC but not insula Orexin-1 receptors promote excessive alcohol drinking. *Front. Neurosci.* 10:400. 10.3389/fnins.2016.00400 27625592 PMC5004043

[B38] Li T. K. Lumeng L. (1984). Alcohol preference and voluntary alcohol intakes of inbred rat strains and the national institutes of health heterogeneous stock of rats. *Alcohol. Clin. Exp. Res.* 8 485–486. 10.1111/j.1530-0277.1984.tb05708.x 6391261

[B39] Lopez M. F. Moorman D. E. Aston-Jones G. Becker H. C. (2016). The highly selective orexin/hypocretin 1 receptor antagonist GSK1059865 potently reduces ethanol drinking in ethanol dependent mice. *Brain Res.* 1636 74–80. 10.1016/j.brainres.2016.01.049 26851547 PMC4808605

[B40] Macey D. J. Schulteis G. Heinrichs S. C. Koob G. F. (1996). Time-dependent quantifiable withdrawal from ethanol in the rat: Effect of method of dependence induction. *Alcohol* 13 163–170. 10.1016/0741-8329(95)02030-6 8814651

[B41] Mahler S. V. Smith R. J. Moorman D. E. Sartor G. C. Aston-Jones G. (2012). Multiple roles for orexin/hypocretin in addiction. *Prog. Brain Res.* 198 79–121. 10.1016/B978-0-444-59489-1.00007-0 22813971 PMC3643893

[B42] Maisel N. C. Blodgett J. C. Wilbourne P. L. Humphreys K. Finney J. W. (2013). Meta-analysis of naltrexone and acamprosate for treating alcohol use disorders: When are these medications most helpful? *Addiction* 108 275–293. 10.1111/j.1360-0443.2012.04054.x 23075288 PMC3970823

[B43] Martin-Fardon R. Weiss F. (2014). N-(2-methyl-6-benzoxazolyl)-N’-1,5-naphthyridin-4-yl urea (SB334867), a hypocretin receptor-1 antagonist, preferentially prevents ethanol seeking: Comparison with natural reward seeking. *Addict. Biol.* 19 233–236. 10.1111/j.1369-1600.2012.00480.x 22830647 PMC3491173

[B44] Martin-Fardon R. Ciccocioppo R. Massi M. Weiss F. (2000). Nociceptin prevents stress-induced ethanol- but not cocaine-seeking behavior in rats. *Neuroreport* 11 1939–1943. 10.1097/00001756-200006260-00026 10884047

[B45] Matzeu A. Martin-Fardon R. (2020). Blockade of orexin receptors in the posterior paraventricular nucleus of the thalamus prevents stress-induced reinstatement of reward-seeking behavior in rats with a history of ethanol dependence. *Front. Integr. Neurosci.* 14:599710. 10.3389/fnint.2020.599710 33240054 PMC7683390

[B46] Matzeu A. Martin-Fardon R. (2021). Understanding the role of orexin neuropeptides in drug addiction: Preclinical studies and translational value. *Front. Behav. Neurosci.* 15:787595. 10.3389/fnbeh.2021.787595 35126069 PMC8811192

[B47] Matzeu A. Kallupi M. George O. Schweitzer P. Martin-Fardon R. (2018a). Dynorphin counteracts orexin in the paraventricular nucleus of the thalamus: Cellular and behavioral evidence. *Neuropsychopharmacology* 43 1010–1020. 10.1038/npp.2017.250 29052613 PMC5854806

[B48] Matzeu A. Terenius L. Martin-Fardon R. (2018b). Exploring sex differences in the attenuation of ethanol drinking by naltrexone in dependent rats during early and protracted abstinence. *Alcohol Clin. Exp. Res.* 42 2466–2478. 10.1111/acer.13898 30320880 PMC6286204

[B49] Mieda M. Yanagisawa M. (2002). Sleep, feeding, and neuropeptides: Roles of orexins and orexin receptors. *Curr. Opin. Neurobiol.* 12 339–345. 10.1016/s0959-4388(02)00331-8 12049942

[B50] Millan E. Z. Furlong T. M. McNally G. P. (2010). Accumbens shell-hypothalamus interactions mediate extinction of alcohol seeking. *J. Neurosci.* 30 4626–4635. 10.1523/JNEUROSCI.4933-09.2010 20357113 PMC6632314

[B51] Moorman D. E. (2018). The hypocretin/orexin system as a target for excessive motivation in alcohol use disorders. *Psychopharmacology (Berl)* 235 1663–1680. 10.1007/s00213-018-4871-2 29508004 PMC5949267

[B52] Moorman D. E. Aston-Jones G. (2009). Orexin-1 receptor antagonism decreases ethanol consumption and preference selectively in high-ethanol–preferring Sprague–Dawley rats. *Alcohol* 43 379–386. 10.1016/j.alcohol.2009.07.002 19671464 PMC2741398

[B53] Moorman D. E. James M. H. Kilroy E. A. Aston-Jones G. (2016). Orexin/hypocretin neuron activation is correlated with alcohol seeking and preference in a topographically specific manner. *Eur. J. Neurosci.* 43 710–720. 10.1111/ejn.13170 26750264 PMC4783285

[B54] Moorman D. E. James M. H. Kilroy E. A. Aston-Jones G. (2017). Orexin/hypocretin-1 receptor antagonism reduces ethanol self-administration and reinstatement selectively in highly-motivated rats. *Brain Res.* 1654(Pt A) 34–42. 10.1016/j.brainres.2016.10.018 27771284 PMC5123944

[B55] National Research Council (2013). *Guidelines for the care and use of mammals in neuroscience and behavioral research.* Washington: National Academy Press.

[B56] O’Dell L. E. Roberts A. J. Smith R. T. Koob G. F. (2004). Enhanced alcohol self-administration after intermittent versus continuous alcohol vapor exposure. *Alcohol Clin. Exp. Res.* 28 1676–1682. 10.1097/01.alc.0000145781.11923.4e 15547454

[B57] Olney J. J. Navarro M. Thiele T. E. (2017). The role of orexin signaling in the ventral tegmental area and central amygdala in modulating binge-like ethanol drinking behavior. *Alcohol Clin. Exp. Res.* 41 551–561. 10.1111/acer.13336 28097729 PMC5332299

[B58] Percie du Sert N. Hurst V. Ahluwalia A. Alam S. Avey M. T. Baker M. (2020). The ARRIVE guidelines 2.0: Updated guidelines for reporting animal research. *BMJ Open Sci.* 4:e100115. 10.1136/bmjos-2020-100115 34095516 PMC7610906

[B59] Peyron C. Tighe D. K. van den Pol A. N. de Lecea L. Heller H. C. Sutcliffe J. G. (1998). Neurons containing hypocretin (orexin) project to multiple neuronal systems. *J. Neurosci.* 18 9996–10015. 9822755 10.1523/JNEUROSCI.18-23-09996.1998 PMC6793310

[B60] Plaza-Zabala A. Flores A. Maldonado R. Berrendero F. (2012). Hypocretin/orexin signaling in the hypothalamic paraventricular nucleus is essential for the expression of nicotine withdrawal. *Biol. Psychiatry* 71 214–223. 10.1016/j.biopsych.2011.06.025 21831361

[B61] Plaza-Zabala A. Flores A. Martin-Garcia E. Saravia R. Maldonado R. Berrendero F. (2013). A role for hypocretin/orexin receptor-1 in cue-induced reinstatement of nicotine-seeking behavior. *Neuropsychopharmacology* 38 1724–1736. 10.1038/npp.2013.72 23518606 PMC3717542

[B62] Richards J. K. Simms J. A. Steensland P. Taha S. A. Borgland S. L. Bonci A. (2008). Inhibition of orexin-1/hypocretin-1 receptors inhibits yohimbine-induced reinstatement of ethanol and sucrose seeking in long-evans rats. *Psychopharmacology (Berl)* 199 109–117. 10.1007/s00213-008-1136-5 18470506 PMC2668563

[B63] Roberts A. J. Cole M. Koob G. F. (1996). Intra-amygdala muscimol decreases operant ethanol self-administration in dependent rats. *Alcohol Clin. Exp. Res.* 20 1289–1298. 10.1111/j.1530-0277.1996.tb01125.x 8904984

[B64] Rosner S. Hackl-Herrwerth A. Leucht S. Lehert P. Vecchi S. Soyka M. (2010). Acamprosate for alcohol dependence. *Cochrane Database Syst. Rev.* CD004332. 10.1002/14651858.CD004332.pub2 20824837 PMC12147086

[B65] Sakurai T. Amemiya A. Ishii M. Matsuzaki I. Chemelli R. M. Tanaka H. (1998). Orexins and orexin receptors: A family of hypothalamic neuropeptides and G protein-coupled receptors that regulate feeding behavior. *Cell* 92 573–585. 10.1016/s0092-8674(02)09256-5 9491897

[B66] Schneider E. R. Rada P. Darby R. D. Leibowitz S. F. Hoebel B. G. (2007). Orexigenic peptides and alcohol intake: Differential effects of orexin, galanin, and ghrelin. *Alcohol Clin. Exp. Res.* 31 1858–1865. 10.1111/j.1530-0277.2007.00510.x 17850217

[B67] Shoblock J. R. Welty N. Aluisio L. Fraser I. Motley S. T. Morton K. (2011). Selective blockade of the orexin-2 receptor attenuates ethanol self-administration, place preference, and reinstatement. *Psychopharmacology (Berl)* 215 191–203. 10.1007/s00213-010-2127-x 21181123

[B68] Sidhpura N. Weiss F. Martin-Fardon R. (2010). Effects of the mGlu2/3 agonist LY379268 and the mGlu5 antagonist MTEP on ethanol seeking and reinforcement are differentially altered in rats with a history of ethanol dependence. *Biol. Psychiatry* 67 804–811. 10.1016/j.biopsych.2010.01.005 20189165 PMC2854322

[B69] Srinivasan S. Simms J. A. Nielsen C. K. Lieske S. P. Bito-Onon J. J. Yi H. (2012). The dual orexin/hypocretin receptor antagonist, almorexant, in the ventral tegmental area attenuates ethanol self-administration. *PLoS One* 7:e44726. 10.1371/journal.pone.0044726 23028593 PMC3448615

[B70] Stephens M. A. Wand G. (2012). Stress and the HPA axis: Role of glucocorticoids in alcohol dependence. *Alcohol Res.* 34 468–483. 23584113 10.35946/arcr.v34.4.11 PMC3860380

[B71] Sun H. Yee K. L. Gill S. Liu W. Li X. Panebianco D. (2015). Psychomotor effects, pharmacokinetics and safety of the orexin receptor antagonist suvorexant administered in combination with alcohol in healthy subjects. *J. Psychopharmacol.* 29 1159–1169. 10.1177/0269881115609015 26464455

[B72] Sutcliffe J. G. de Lecea L. (2000). The hypocretins: Excitatory neuromodulatory peptides for multiple homeostatic systems, including sleep and feeding. *J. Neurosci. Res.* 62 161–168. 10.1002/1097-4547(20001015)62:2<161::AID-JNR1<3.0.CO;2-1 11020209

[B73] Teske J. A. Billington C. J. Kotz C. M. (2010). Hypocretin/orexin and energy expenditure. *Acta Physiol. (Oxf)* 198 303–312. 10.1111/j.1748-1716.2010.02075.x 20070282

[B74] Torres O. V. Walker E. M. Beas B. S. O’Dell L. E. (2014). Female rats display enhanced rewarding effects of ethanol that are hormone dependent. *Alcohol Clin. Exp. Res.* 38 108–115. 10.1111/acer.12213 23909760 PMC3842413

[B75] Uslaner J. M. Winrow C. J. Gotter A. L. Roecker A. J. Coleman P. J. Hutson P. H. (2014). Selective orexin 2 receptor antagonism blocks cue-induced reinstatement, but not nicotine self-administration or nicotine-induced reinstatement. *Behav. Brain Res.* 269 61–65. 10.1016/j.bbr.2014.04.012 24746488

[B76] Vendruscolo L. F. Roberts A. J. (2014). Operant alcohol self-administration in dependent rats: Focus on the vapor model. *Alcohol* 48 277–286. 10.1016/j.alcohol.2013.08.006 24290310 PMC4007394

[B77] von der Goltz C. Koopmann A. Dinter C. Richter A. Grosshans M. Fink T. (2011). Involvement of orexin in the regulation of stress, depression and reward in alcohol dependence. *Horm. Behav.* 60 644–650. 10.1016/j.yhbeh.2011.08.017 21945150

[B78] Walker B. M. Walker J. L. Ehlers C. L. (2008). Dissociable effects of ethanol consumption during the light and dark phase in adolescent and adult Wistar rats. *Alcohol* 42 83–89. 10.1016/j.alcohol.2007.12.001 18358986 PMC2387129

[B79] Walker L. C. Lawrence A. J. (2017). The role of orexins/hypocretins in alcohol use and abuse. *Curr. Top. Behav. Neurosci.* 33 221–246. 10.1007/7854_2016_55 27909991

[B80] Winsky-Sommerer R. Yamanaka A. Diano S. Borok E. Roberts A. J. Sakurai T. (2004). Interaction between the corticotropin-releasing factor system and hypocretins (orexins): A novel circuit mediating stress response. *J. Neurosci.* 24 11439–11448. 10.1523/JNEUROSCI.3459-04.2004 15601950 PMC6730356

[B81] Witkiewitz K. Litten R. Z. Leggio L. (2019). Advances in the science and treatment of alcohol use disorder. *Sci. Adv.* 5:eaax4043. 10.1126/sciadv.aax4043 31579824 PMC6760932

[B82] Zhao Y. Dayas C. V. Aujla H. Baptista M. A. Martin-Fardon R. Weiss F. (2006). Activation of group II metabotropic glutamate receptors attenuates both stress and cue-induced ethanol-seeking and modulates c-fos expression in the hippocampus and amygdala. *J. Neurosci.* 26 9967–9974. 10.1523/JNEUROSCI.2384-06.2006 17005860 PMC6674480

[B83] Ziolkowski M. Czarnecki D. Budzynski J. Rosinska Z. Zekanowska E. Goralczyk B. (2016). Orexin in patients with alcohol dependence treated for relapse prevention: A pilot study. *Alcohol Alcohol.* 51 416–421. 10.1093/alcalc/agv129 26597795

